# How Does Social Insurance Affect the Social Interactions of Rural Residents in China: Study on the Impact of Rural Formal Social Security System on Informal Social Security Mechanism

**DOI:** 10.3389/fpsyg.2022.751946

**Published:** 2022-03-09

**Authors:** Ming Zhang, Lan Yuan, Zhanlian Ke, Juanfeng Jian, Hong Tan, Gangwu Lv

**Affiliations:** ^1^College of State Governance, Southwest University, Chongqing, China; ^2^School of Public Policy and Administration, Chongqing University, Chongqing, China; ^3^Chongqing Youth and Vocational Technical College, Chongqing, China; ^4^College of Resources and Environment, Southwest University, Chongqing, China

**Keywords:** rural social insurance, social networks, propensity score matching method, Chinese General Social Survey, grouping test

## Abstract

With the increasing mobility of the rural population in China and the growing number of residents moving to the cities for work or study, rural society is forming a pluralistic, interest-centered, “open” social networks relations that follows the modern rule of law contract. Based on Chinese General Social Survey (CGSS) data, the results of the empirical study finds that social insurance can significantly enhance the social interactions of rural residents in China, that is, formal social security system in rural areas promotes informal social security mechanisms such as social interactions. The results of the grouping test show that rural residents in China’s different regions were affected by social insurance in different ways. Social insurance has a greater impact on the social interactions in the eastern region than in the middle and western regions. The propensity score matching method was used to reduce the sample selection bias, and the findings of the paper were found to be robust.

## Introduction

From the strategic goal of “coordinating and promoting the construction of urban and rural social security system, and fully completing a social security system covering urban and rural residents by 2020” proposed at the 18th congress, to the strategic goal of “improving the basic pension insurance system of urban and rural residents (BPISURR)” and “improving the basic medical insurance system for urban and rural residents (BMISURR)” proposed at the 19th congress, it highlights the importance of social security system in current Chinese society. In this condition, China has been improving the rural social security system in recent years, forming a social security system in rural areas with the urban and rural basic pension insurance system and the new rural cooperative medical system as the core. At present, the number of people participating in urban and rural basic pension insurance is increasing, and the participation rate of new rural cooperative medical care has reached more than 98%, so the overall development of rural social insurance is good. As an important welfare system, the traditional perspective of social insurance focuses more on the income redistribution effect it brings, but a large number of studies have shown that social insurance also creates complex socioeconomic impact by affecting consumption, labor market and human capital.

Although a large number of studies have found that the impact of social insurance on residents’ consumption depends on factors such as funding patterns and willingness to save (Gormley et al., 2010; Van, 2012, 2016; Alessie et al., 2013; Wang Z., 2014), recent studies have shown that social insurance has a significant positive effect on residents’ consumption for both developed and developing countries (Aydede, 2007; Ji and Zhao, 2013; Wang Y., 2014; Wang, 2018; Chen et al., 2015; Tian et al., 2016; Fan et al., 2017; Zhang et al., 2017; Shi and Zhou, 2021). In terms of its impact on the labor market, social insurance affects not only the willingness to participate in the labor force and the cross-regional mobility of the labor force (Yang, 2003; Hipp, 2016; Bonnet et al., 2018; Ge, 2018; Lapeyre et al., 2020), but also the quality of the workforce, and human capital inputs (Ehrlich and Lui, 1998; Barbie et al., 2006; Wu, 2006; Jia et al., 2011; Zou, 2021).

Social insurance is a system of national livelihood security and social stability with social welfare characteristics, and is a formal welfare system confirmed through legal policies. Obviously, for rural residents, social insurance can guarantee the basic livelihood of rural society members through national income redistribution, thus improving the welfare level of the whole society. However, China is a typical human feeling society, especially in rural areas, villagers usually integrate their relatives, friends, colleagues and other resources to cope with all kinds of difficulties, and social interactions can serve as an informal mechanism for the allocation of resources and information to provide security for residents (Li and Chen, 2012; Yi et al., 2012; Shen and Jia, 2016; Sun and Feng, 2021). Thus, along with the improvement of the rural social security system, will there be an impact on the traditional Chinese rural social networks of relationships based on blood and geographical ties as the cornerstone? In other words, do rural formal social security systems and informal social security mechanisms, represented by social interactions, reinforce each other or do they substitute? The answer to the question is of great practical importance for peeping and understanding rural social relations and governance in the new era.

On the basis of summarizing the characteristics of the rural social relationship networks in the new era, this study will analyze the complex influence mechanism of social insurance on the construction process of new rural social relationship networks. Further based on the 2013 Chinese General Social Survey (CGSS, 2013) data, using the ordinary least squares and propensity score matching methods, the effect of social insurance on rural residents’ social interactions and group differences in this effect are examined empirically. The rest of the paper is organized as follows: the second part reveals the characteristics of the new rural social relationship networks and theoretically analyzes the mechanism of social insurance’s influence on it; the third part introduces the data sources and the design of relevant variables for the empirical study; the fourth part empirically tests the specific impact performance of social insurance on the social interactions of rural residents and examines differences by regional grouping; the fifth part is a robustness test based on the propensity score matching method; and finally concludes the full paper and makes policy recommendations.

## The Characteristics of the New Rural Social Interactions and the Impact of Social Insurance

### Characteristics of the New Rural Social Interactions

Since the reform and opening up, China’s economy and society have undergone radical changes, and the implementation of the household contract responsibility system, the establishment of a socialist market economy system, the construction of a new socialist countryside and the recently proposed rural revitalization strategy have profoundly affected the network of social relations in China’s rural areas. With the increasing mobility of the rural population, the number of villagers moving to the cities for work or study is gradually increasing (Ye, 2007, 2016; Lu, 2011; Wang, 2021), the social networks of villagers is also getting richer, and the rural social interactions has shifted from the traditional single, emotion-centered, “closed” characteristic of following traditional ritual ethics to an “open” social interactions, the basic features are as follows.

### Pluralistic Social Interactions

After the reform and opening up in China, a large number of rural laborers moved to the cities, breaking the closed status of traditional rural society, and the importance of kinship and geo-relations in the rural social interactions has declined, while working relations have become increasingly important (Li, 2013). At the same time, some new social networks have also started to emerge, such as academic relations, marriage relations (Xia, 2014). This is mainly manifested in the fact that a large number of rural household members have gone out to study or work and come into contact with different circles or industries, while villagers’ marriage relations are no longer limited to their own village or surroundings. In addition, along with information technology development and continuous improvement of rural network infrastructure construction in China, most villagers now have access to the Internet, and the development of online virtual communities has greatly broadened the scope of villagers’ social interactions (Cai, 2014; Chen, 2014; Wan et al., 2019).

### Interest-Centered Social Interactions

With the development of market economy in China, market factors such as production efficiency and monetary exchange have profoundly influenced rural social relationship networks, and villagers are increasingly behaving as rational economic man who take maximizing personal interests as their ultimate goal, and interests gradually replace emotions as the dominant factor in rural social relationship networks (He, 2001; Sun, 2016). On the one hand, it is reflected in the change of rural production and operation methods. In order to seek better development, a large number of rural laborers go out to work, and when there is a shortage of rural laborers in the busy farming season, villagers tend to hire other laborers to help, so that the paid market exchange replaces the old unpaid mutual benefit (He, 2012). On the other hand, it is reflected in the rural social life, where the purely emotional communication between villagers is becoming less and less, and more and more is a human relationship integrated with personal interests (Sun, 2016).

### A Social Interactions Following the Modern Rule of Law Contract

Traditional rural social society mainly relies on clan elders to maintain, morality is the main force to judge behavior, while in modern rural social society, the force is the law, China’s rural areas have shifted from traditional ritualistic society to modern rule of law society (Wang, 2009, 2015; Yu, 2014; Shi et al., 2016; Sun and Ren, 2018; Zhang, 2020). With the continuous improvement of the legal system and the increasing legal awareness of villagers, rural residents will regulate the social relationship networks based on the law. For example, in a lending relationship, rural areas used to decide who to lend money to and whether to lend based on trust and familiarity, but now even two people who do not know each other can establish a lending relationship as long as both parties have signed a loan contract (Len and Xiong, 2020). Similarly, labor relations and employment relations can be formed. Thus, a society governed by the rule of law actually reduces the cost of people’s social interactions and helps broaden the network of social relationship among people.

### The Mechanism of Social Insurance Affecting Social Interactions of Rural Residents

Currently, in China, with the implementation of a series of policies to support agriculture, the overall income level of rural residents has increased to a considerable extent, but the gap between high-income and low-income earners is widening. Social insurance, as a social welfare system and income redistribution policy, can increase the disposable income of rural low-income groups (Zhang and Ma, 2009; Zheng, 2010; Wang and Xue, 2012; Liao et al., 2015; Zhang et al., 2015; Wang et al., 2016; Deng and Bu, 2019; Yu and Ma, 2021; Zhao, 2021), not only does it increase the amount of money spent on social networks maintenance, but it also supports rural residents’ access to education and the expansion of their networks based on “academic” relationship. Meanwhile, social insurance can also increase rural residents’ sense of social fairness (Zhu, 2010; Yang and Zhang, 2016; Yu and Zhang, 2018), improve their social trust and thus increase their willingness to socialize. With the improvement of social insurance policies, it is also likely to have an alternative function to the informal security system of social interactions.

### Social Insurance Sustains Social Interactions by Mitigating Income Uncertainty and Increasing Gift Expenditure

In current China’s rural society, gift expenditure is an important way to sustain social interactions (Hu and Wei, 2013; Zhou and Ma, 2015; Yan et al., 2016; Ma and Ding, 2018; Fu, 2021). Due to the relatively low-income level of rural residents in China, gift expenditures often bring greater financial pressure to them. In particularly, the rising gift expenditure in recent years has increased the burden of villagers’ livelihood. Social insurance increases villagers’ expected future income to a certain extent, and it enables them to continue to maintain or even increase their interaction and thus strengthen their rural social interactions.

### Social Insurance Weakens Social Interactions by Improving Uncertainty Expectations

The most important features of traditional rural social relations in China are reciprocity, mutual aid and trust (Zhang and Zhao, 2016). Posner (1980) argues that mutual aid behavior among people comes from rational choices made in anticipation of environmental and risk uncertainty. Before the formation of the rural social security system, villagers had to face getting old or ill, natural disasters and various other risks. In order to reduce the uncertainty of the future, they trusted each other, cooperated with each other, established social interactions of mutual help and reciprocity, and formed an interactive mechanism to maintain the network of relationships, i.e., human interaction. However, the establishment and improvement of rural social insurance has now ensured the basic livelihood of villagers and alleviated the uncertainty expectations of rural society members (Gan et al., 2010; Ma and Zhang, 2011; Li, 2014; Liu and Xie, 2017; Wang and Li, 2021). Especially in today’s rural areas in China, the alienation of human relations appears, and human relations often become a tool for enrichment (Chen, 2011; He, 2011, 2014), so the establishment of a social insurance system has discouraged villagers from spending more on maintaining rural social relations. Moreover, with the continuous improvement of the rural pension insurance system, the concept of raising children for getting old has been impacted, the family protection function has diminished, and the traditional blood-based social relationship networks have been weakened as the ancestor-grandson relationship, kinship relationship and even father-son relationship are gradually alienated.

### Data and Research Methods

The data for the empirical analysis are obtained from the 2013 Chinese General Social Survey (CGSS, 2013), which comprehensively collects data at society, community, family, and individual levels in order to summarize the trends of social change in China. This paper focuses on the impact of social insurance on rural social interactions in China, removing survey respondents with missing data and including a total sample of 5,201 respondents. The design of the main variables of the empirical study is presented below.

Social network refer to all formal and informal social bonds that individuals form with other people, including both direct social relationship with people and indirect relationship formed through sharing physical environment and culture (Mitchell, 1969). The group nature of human social life determines that people are bound to form various relationships with each other, and it mainly consists of relatives, friends, colleagues or neighbors. The study uses the frequency of social interactions as a cohesion characterization scale to characterize rural residents’ social interactions (*Sinter*). We selected five questions related to the frequency of social interactions as the base indicators according to the questionnaire design of CGSS (2013), and the indicator selection and numerical design are shown in the Table 1.

**TABLE 1:** Basic indicators of villagers’ social relationship networks.

Factor	Problems	Numerical design
A30.6	In the past year, how often have you engaged in the following activities in your free time: meeting with relatives?	“Never” = 1; “Several times a year or less” = 2; “Several times a month” = 3; “Several times a week” = 4; “Every day” = 5
A30.7	In the past year, how often have you engaged in the following activities in your free time: partying with friends?	“Never” = 1; “Several times a year or less” = 2; “Several times a month” = 3; “Several times a week” = 4; “Every day” = 5
A31.1	In the past year, how often have you done the following things in your free time: socializing/going out?	“Never” = 1; “rarely” = 2; “sometimes” = 3; “often” = 4; “very frequent” = 5
A31a	How often do you and your neighbors engage in social entertainment activities (such as visiting each other, watching TV together, eating, playing cards)	“Never” = 1; “1 time a year or less” = 2; “Several times a year” = 3; “About 1 time a month” = 4; “Several times a month” = 5; “1 to 2 times a week” = 6; “almost every day” = 7
A31b	How often do you and your friends engage in social entertainment activities (such as visiting each other, watching TV together, eating, playing cards)	“Never” = 1; “1 time a year or less” = 2; “Several times a year” = 3; “About 1 time a month” = 4; “Several times a month” = 5; “1 to 2 times a week” = 6; “almost every day” = 7

The selected indicators were standardized, and the social relationship networks was measured by principal component analysis based on Stata software. Where a negative value indicates that the social interactions of the sample individuals is lower than the average of the overall social interactions of the sample, and a positive value vice versa.

This paper selects several questions on social security in CGSS (2013) to observe villagers’ social insurance participation. The answer to the question “Are you enrolled in BMISURR” is *Security1*, and the answer to the question “Are you enrolled in BPISURR” is *Security2*. The BMISURR and the BEISURR are the main forms of social insurance existing in rural areas, therefore we mainly use *Security1* and *Security2* as indicators to reflect social insurance. The social security section of the CGSS also covers two other questions, more in the commercial sense of the insurance coverage approach. The question “Are you enrolled in commercial medical insurance (CMI)” is *Security3*, and the question “Are you enrolled in commercial pension insurance (CPI)” is *Security4*. For *Security1*, *Security2*, *Security3* and *Security4*, the value of 1 is assigned when the resident selects “Participate” and 0 is assigned for “Doesn’t Participate.”

The existing studies found that the inclination of social interactions, education level, social equality and uncertainty expectations were important factors which can influence social interactions (Jia et al., 2011; Liu and Xie, 2017; Wang, 2017; Ma and Ding, 2018), and the study try to estimate it. In the “Behavior and Attitudes” section of the CGSS, the self-assessment question on inclination toward social interaction (*inclination*) is “I am always reluctant to associate with people who are not in a good position to live,” and we assigned values of 1, 2, 3 and 4 to the question when residents answered “very much in line with,” “more in line with,” “not quite in line with,” and “very much out of line,” respectively. For educational level (*edu*), we obtained it based on the answer to the question “What is your current highest level of education?”. When the respondents’ education level was illiterate, elementary school, junior high school, high school (junior college), and college and above, it was assigned from 1 to 5, respectively. On the measurement of perceived social equality (*equality*) was taken directly from the CGSS questionnaire, “In general, do you think society is fair or unfair today?”. The answers to this question were assigned a value of 1-5 when respondents answered “not fair at all,” “relatively unfair,” “not fair but not unfair either,” “Fairly fair” and “Completely fair.” Since the risk of care for the elderly is an important dimension of uncertainty expectations in China, this study uses the CGSS questionnaire to characterize the uncertainty expectations of residents by their perceptions of the issue of “lack of security in old age.” In this study, when the respondents perceive the situation as “very less serious,” “less serious,” “moderate,” “more serious,” and “very serious,” the valve of uncertainty is assigned as 1, 2, 3, 4, 5, respectively.

To develop the econometric model analysis, the study further controlled other variables that affect social interactions. The indicator selection and numerical design are shown in the Table 2.

**TABLE 2:** Main variables affecting social networks.

Name	Question	Numerical design
Security1	Are you enrolled in BMISURR?	“Participate” = 1; “Doesn’t Participate” = 0
Security2	Are you enrolled in BPISURR?	
Security3	Are you enrolled in commercial medical insurance (CMI)?	
Security4	Are you enrolled in commercial pension insurance (CPI)?	
Inclination	I am always reluctant to associate with people who are not in a good position to live.	When residents answered “very much in line with,” “more in line with,” “not quite in line with,” and “very much out of line,” it was assigned from 1 to 4, respectively.
Edu	What is your current highest level of education?	When the respondents’ education level was illiterate, elementary school, junior high school, high school (junior college), and college and above, it was assigned from 1 to 5, respectively.
Equality	In general, do you think society is fair or unfair today?	When respondents answered “not fair at all,” “relatively unfair,” “not fair but not unfair either” “Fairly fair” and “Completely fair,” it was assigned from 1 to 5, respectively.
Uncertainty	Lack of security in old age.	When the respondents perceive the situation as “very less serious,” “less serious”, “moderate”, “more serious”, and “very serious,” it was assigned from 1 to 5, respectively.
Age	The age of the respondent in 2013.	The answers are the values provided by the respondents.
Gender	The gender of the respondent in 2013.	“Male” = 1; “Female” = 0
Morality	The morality of the respondent in 2013.	“Han nationality” = 1; “Ethnic minorities” = 0
Politic	The politic state of the respondent in 2013.	“Party members” = 1; “Non-Party members” = 0
Health	What do you think of your health status?	When the respondents choose “very unhealthy,” “relatively unhealthy,” “average,” “relatively healthy” and “very healthy,” it was assigned from 1 5, respectively.
Marriage	The marriage state of the respondent in 2013.	“cohabiting” and “married” = 1; the other = 0
Work	What is your work experience and status?	“currently working in non-farm work” = 1; the other = 0
Income	What was your total personal income for the whole of last year (2012)?	The answers are the values provided by the respondents.
House	The number of respondents’ houses.	The answers are the values provided by the respondents.

Table 3 reports the descriptive statistics of the main variables involved in this paper, with a total sample of 5201, of which 90.8% participated in the BMISURR, 62.8% participated in the BPISURR, 4.8% participated in the CMI, and 3.2% participated in the CPI.

**TABLE 3:** Descriptive analysis of main variables.

Variable	Mean	SD	Min	Max
Sinter	–0.091	0.654	–1.559	2.150
Secruity1	0.908	0.256	0	1
Secruity2	0.628	0.473	0	1
Secruity3	0.048	0.208	0	1
Secruity4	0.032	0.181	0	1
Inclination	2.917	1.571	1	4
Edu	3.593	2.357	1	5
Equality	3.901	1.881	1	5
Uncertainty	3.905	1.793	1	5
Age	48.356	15.922	17	91
Gender	0.501	0.485	0	1
Morality	0.893	0.310	0	1
Politic	0.047	0.216	0	1
Health	3.668	1.132	1	5
Marriage	0.819	0.364	0	1
Work	0.521	0.324	0	1
Income	1.583	3.031	0	80
House	1.096	0.504	0	10

*The total number of observation is 5201.*

## Preliminary Analysis Results

### Basic Estimation Results

In this paper, the indicator values of the rural social interactions were obtained by principal component analysis, and the OLS method was next used to test the effect of social insurance on the social interactions of rural residents in China. The related regression results are reported in Table 4. Model (1) in Table 4 reports the results of regressions with respondents’ social interactions (*Sinter*) as the explanatory variable, incorporating the four variables of social security, and controlling for the age variable. Four variables of social security (*Secruity1*, *Security2*, *Security3*, *Secruity4*) have significantly positive estimated coefficients. After further controlling for other influencing variables and individual characteristic variables, the estimated coefficients of *Secruity1* and *Security2* in model (2) and model (3) are still significantly positive, but the estimated coefficients of *Security3* and *Secruity4* are less significant and even become insignificant. This suggests that government-led welfare social insurance is more likely to enhance residents’ social interactions than private insurance.

**TABLE 4:** The effect of social insurance on rural residents’ social interactions.

	(1)	(2)	(3)
Security1	0.169 (0.024)***	0.153 (0.040)***	0.151 (0.045)***
Security2	0.077 (0.023)***	0.057 (0.022)***	0.050 (0.013)***
Security3	0.155 (0.047)***	0.121 (0.069)*	0.109 (0.057)*
Security4	0.146 (0.070)**	0.084 (0.071)	0.099 (0.076)
Age	–0.044 (0.007)***	–0.043 (0.008)***	–0.031 (0.010)***
Age^2^	0.032 (0.004)***	0.024 (0.006)***	0.015 (0.005)***
Inclination		0.021 (0.007)***	0.024 (0.006)***
Edu		0.032 (0.008)***	0.028 (0.005)***
Equality		0.070 (0.025)***	0.062 (0.015)***
Uncertainty		0.038 (0.022)*	0.041 (0.034)
Gender			–0.012 (0.009)
Morality			–0.061 (0.022)***
Politic			0.174 (0.041)***
Health			0.085 (0.022)***
Marriage			–0.072 (0.016)***
Work			0.038 (0.017)**
Income			0.018 (0.007)**
House			0.070 (0.024)***
Province	control	control	control
*R*^2^ or pseudo *R*^2^	0.110	0.086	0.111

*, **, *** mean significant in 10%, 5%, and 1% level, respectively, standard errors are in parentheses, and the number of observation is 5201.

The estimated coefficients of the *inclination* are significantly positive, which is consistent with reality of the inclination of social interactions is an important way to maintain its intensity. The estimated coefficient of the *edu* are significantly positive, and it implies that the higher the level of education, the richer the social interactions of rural residents, which is easy to understand because education increases the social interactions based on “schooling.” The coefficients of *equality* are also significantly positive, and it means that an increased sense of equality can increase residents’ willingness to socially interact, thus enhancing the social interactions of rural residents. The coefficients of *uncertainty* are positive but insignificant, which indicates that in China’s rural areas, due to the inadequate formal security system, residents have the motive to fight against uncertainty through informal security such as “relationship,” but this functional motive is weakening (He, 2001; Sun, 2016).

The estimated coefficient of the *age* in Table 4 is negative, but the coefficient of age^2^ is significantly positive, indicating a “U-shaped” change between residents’ age and social interactions. However, combined with the inflection point of “U-shaped” curve, we can find that the age of our respondents is on the right side of the inflection point of the “U-shaped” curve. So there is a positive relationship between the social interactions of residents and their age, and the older the residents are, the richer their social interactions is. The estimated coefficient of *gender* is insignificant, indicating that there is insignificant difference in social interactions between men and women in rural areas; the coefficient of *morality* is significantly negative, indicating that the social interactions of ethnic minority villagers are stronger than those of Han nationality. The mainly reason is that ethinc groups habitation is similar to small and marginalized community, and ethnic minority villagers interact more in their community as they have access less to the bigger network due to the differences in customs and traditions. the coefficient of *politic* is significantly positive, indicating that the social interactions of Party members are richer than those of non-Party members; the coefficient of *health* is significantly positive, because healthy people will devote more energy to social interactions; the coefficient of *marriage* is significantly negative, indicating that unmarried people have more time and energy to devote to social interaction; the estimated coefficient of *work* is significantly positive, and it means that farmers who are engaged in non-farm work have more frequent social interactions, which indicates that non-farm work such as “part-time work” has expanded the social interactions of rural residents based on job; the estimated coefficient of *income* is significantly positive, indicating that the higher the income, the stronger the social interactions; the estimated coefficient of *house* is significantly positive, indicating that the more the number of properties are, the stronger the social interactions is.

The results in Table 4 show that government-led welfare social insurance has an positive impact on residents’ social interactions. It means that the establishment of a formal social security system in rural areas has facilitated informal social security mechanisms such as social interactions. According to previous analysis, it may result from its effects on easing budget constraints. In addition, the results imply that the mitigating effect of social insurance on residents’ uncertainty is not obvious at this stage. This is likely due to the fact that the formal protection system, such as rural pension insurance, is still being gradually constructed and improved, and thus the psychological expectation of residents to reduce uncertainty by participating in social insurance has not yet been formed.

### Regional Grouping Estimation Results and Analysis

Along with reform measures such as the household contract responsibility system in China, the establishment of the socialist market economy system, the construction of the new socialist countryside, and the recently proposed rural revitalization strategy, the social life of rural residents has changed dramatically (Ma and Jia, 2017). Meanwhile, the accentuation of income disparity between regions have made the social life of rural residents in different regions different. Next, this study is grouped based on region in order to explore the impact of social insurance on the social interactions of rural residents in different regions, with different incomes. Since there are significant differences between the eastern and the middle and western region of China in terms of both the degree of economic development and the process of market-oriented reform, the development of rural social insurance also differs greatly, so the study discusses the differences of impact of social insurance on social network between the eastern and the mid-western region. According to the criteria for dividing the eastern, middle and western region in the China Statistical Yearbook and the China Health Statistical Yearbook, 11 provinces including Beijing, Tianjin, Hebei, Liaoning, Shanghai, Jiangsu, Zhejiang, Fujian, Shandong, Guangdong and Hainan are classified as the eastern region, while the remaining provinces are classified as the mid-western region. Based on the OLS estimation method, the relevant empirical results are reported in Table 5. Model (4) and (5) in Table 5 show the estimation results for the eastern region sample, while models (6) and (7) show the corresponding estimation results for the mid-western region. The estimated coefficients of both *Secruity1* and *Security2* are significantly positive, which is consistent with the overall sample. However, comparing the estimated results between the eastern and the mid-western region, we find that the estimated coefficients of *Security1* and *Security2* are higher in the eastern region than in the mid-western region, which indicates that the social insurance has a greater role in promoting the social relationship network of villagers in the eastern region. It also implies that in areas with higher levels of economic development and marketization, the contribution of the formal rural security system to informal security mechanisms is more pronounced. Among the control variables, the estimated coefficients *income*, *house*, *politic*, and *morality* are significant in the sample from the mid-western region, while the estimated coefficients of those variables are not significant in the eastern region. The estimation results of other variables are consistent with Table 3.

**TABLE 5:** Grouping test of Villagers in Eastern and Mid-western region.

	Eastern region		Mid-western region	
	
	(4)	(5)	(6)	(7)
Security1	0.212 (0.063)***	0.219 (0.070)***	0.175 (0.052)***	0.169 (0.050)***
Security2	0.142 (0.050)***	0.132 (0.034)***	0.074 (0.024)***	0.070 (0.033)**
Security3	0.186 (0.094)*	0.120 (0.091)	0.156 (0.070)**	0.075 (0.062)
Security4	0.081 (0.111)	0.063 (0.145)	0.138 (0.076)*	0.086 (0.054)
Age	–0.041 (0.014)***	–0.037 (0.012)***	–0.040 (0.008)***	–0.031 (0.008)***
Age^2^	0.021 (0.010)**	0.034 (0.025)	0.032 (0.007)***	0.026 (0.010)***
Inclination		0.031 (0.006)***		0.027 (0.007)***
Edu		0.026 (0.025)		0.026 (0.007)***
Equality		0.070 (0.017)***		0.054 (0.017)***
Uncertainty		0.034 (0.025)		0.033 (0.031)
Gender		0.015 (0.025)		–0.012 (0.016)
Morality		–0.046 (0.052)		–0.115 (0.040)***
Politic		0.141 (0.102)		0.183 (0.054)***
Health		0.077 (0.021)***		0.070 (0.022)***
Marriage		–0.116 (0.063)*		–0.125 (0.041)**
Work		0.032 (0.011)**		0.030 (0.015)**
Income		0.011 (0.013)		0.008 (0.004)**
House		0.059 (0.044)		0.052 (0.017)***
Province	control	control	control	control
*R*^2^ or pseudo *R*^2^	0.213	0.234	0.134	0.142
Observation	1108	1108	4093	4093

*, **, *** indicate significant in 10%, 5%, and 1% levels, respectively, and the standard errors are in parentheses.

### Propensity Score Matching Estimation Analysis

Because of the obvious individual heterogeneity between villagers who participate in social insurance and those who do not, there are obvious differences in terms of individual characteristics such as age, income, education status, marital status, social class, and political 0rientation. As a consequence, a direct regression analysis using the OLS method will produce a certain degree of sample selection bias. Since it is infeasible to directly observe the social interactions status of the same villagers with and without social insurance, the Propensity score matching method proposed by Rosenbaum and Rubin (1985) is used to reduce the sample selection bias. This is done by matching villagers who are covered by social insurance (enrolled in either) (called the treatment group) with villagers who are not covered by social insurance (called the control group) and estimating the average treatment effect. In this study, several common matching methods are used to match the treatment and control groups, including the nearest neighbor matching method, radius matching method, kernel matching method and local linear matching method. Nearest neighbor matching method is the most commonly used matching method. Using the nearest neighbor matching method, the sample mean treatment effect was 0.1404 (see Table 6). Although there are slight differences in the mean treatment effects among radius matching method, kernel matching method, and local linear matching method, those are all significantly positive at the near 1% level, suggesting that social insurance still enhances villagers’ social interactions after reducing the sample selection bias.

**TABLE 6:** Propensity score matching estimation results.

PSM	Treatment group	Control group	Average treatment effect	Robust	T
The nearest neighbor matching method	–0.0733	–0.2135	0.1402	0.0383	3.66***
Radius matching method	–0.0754	–0.1874	0.1120	0.0431	2.60***
Kernel matching method	–0.0814	–0.2535	0.1721	0.0498	3.46***
Local linear matching method	–0.0788	–0.2272	0.1484	0.0531	2.79***

*** indicate significant in 1% level.

To ensure the robustness of the estimation results of the propensity score matching method, the sample is next tested for equilibrium. The purpose of the equilibrium test is to ensure that after matching, there are no significant differences in the variables except for differences in the key explanatory variables. We tested the matching equilibrium by calculating the standard deviation of each matching variable between participating and non-participating social insurance after pairing. It is generally believed that, as long as the absolute value of standard deviation is less than 20, it will not cause the failure of matching (Rosenbaum and Rubin, 1985), and the smaller the value of standard deviation is, the better the model matching effect is. Table 7 reports the standard deviations of each matched variable which are significantly less than 20, and the *t*-test could not reject the original hypothesis of no significant difference in the matched variables at the 10% significance level. So it satisfies the requirement of matching equilibrium and indicates that we obtained more robust estimation results using propensity score matching methods.

**TABLE 7:** Results of balance test.

	Mean of treatment group	Mean of control group	Standard deviation (%)	Standard deviation reduction (%)	Mean of treatment group
Inclination	2.914	2.846	3.4	78.8	0.320
Edu	3.523	3.582	–1.9	81.0	0.521
Equality	4.011	4.036	–1.2	77.4	0.623
Uncertainty	3.825	3.805	0.9	90.3	0.745
Age	47.334	47.45	–1.1	84.2	0.639
Gender	0.514	0.505	2.0	78.3	0.491
Morality	0.832	0.827	0.6	84.2	0.768
Politic	0.048	0.051	–0.8	92.3	0.751
Health	3.669	3.670	–0.9	90.3	0.746
Marriage	0.835	0.791	2.3	77.6	0.373
Work	0.523	0.516	2.3	77.5	0.376
Income	1.612	1.605	1.3	88.7	0.556
House	1.093	1.104	–1.1	83.9	0.675

Next, grouping tests were conducted. In order to verify the differences in the impact of social insurance on villagers’ social interactions in different regions, the paper uses the propensity score matching method to estimate it separately for the eastern and central-western region, and the related estimation results are reported in Table 8. Among the four matching methods, the average treatment effects were significantly positive in both the eastern and mid-western region, and the average treatment effect values were larger in the eastern region than in the mid-western region, indicating that the promotion of social interactions by social insurance was more prominent in the eastern region, which was consistent with the OLS model estimation results.

**TABLE 8:** The matching estimation results of eastern and central and western villagers’ propensity score.

PSM	Group	Treatment group	Control group	Mean treatment effect	Robust	T
The nearest neighbor matching method	East	–0.1141	–0.3266	0.2125	0.0824	2.58**
	Mid-west	–0.0643	–0.1639	0.0996	0.0530	1.88*
Radius matching method	East	–0.1240	–0.3755	0.2515	0.0717	3.51***
	Mid-west	–0.0893	–0.2037	0.1144	0.0509	2.25**
Kernel matching method	East	–0.1243	–0.3314	0.2071	0.0848	2.44**
	Mid-west	–0.0821	–0.1961	0.1140	0.0515	2.21**
Local linear matching method	East	–0.1227	–0.3278	0.2051	0.0855	2.40**
	Mid-west	–0.0833	–0.2147	0.1314	0.0524	2.51**

*, **, *** indicate significant in 10%, 5%, and 1% level, respectively.

## Conclusion and Policy Recommendations

Along with the increasingly frequent population movement in rural areas and the gradual increase of villagers going to urban areas for work or study in China, the social relationship networks of villagers in China has become richer and richer, and the social relationship networks in rural areas has shifted from the traditional single, emotion-centered, “closed” characteristic of following traditional ritual ethics, to a pluralistic, interest-centered, “open” characteristic of following modern rule of law contract. Based on CGSS (2013) data, the empirical study finds that social insurance can significantly enhance villagers’ social interactions in China, i.e., a formal social security system in rural areas promotes informal social security mechanisms such as social interactions. The results of the grouping tests showed that villagers in different regions were affected by social insurance in different roles. The impact of social insurance on villagers’ social interactions is greater in the eastern region than in the mid-western regions. The propensity score matching method was used to reduce the sample selection bias, and it shows that our results are robust.

The empirical study in this paper helps us to comprehensively understand the profound impact on rural economy and society brought by the process of establishing social security in rural areas, and reminds us that, in the process of cultivating and establishing new social relationship networks in rural areas, the correct and rational use of the positive role of social insurance and social security also requires the following points to be done.

On one hand, the role of social networks as social capital is becoming increasingly important, not only in creating a harmonious interpersonal atmosphere, but also in providing people with information on various aspects of life, employment, and skills. In order to enhance social interactions, it is necessary to ensure that social insurance benefits are truly available to rural people in need, and effectively alleviate the uncertain income expectations of this group. And only in this way can social insurance truly improve the sense of social equity and trust of residents. On the other hand, China’s rural social security system lags behind that of urban areas, and the construction of social security in rural areas of central and western regions lags behind that of eastern regions. Therefore, establishing a sound social insurance system that benefits more regions and more residents will help increase the number of beneficiaries of China’s social security system, thus giving greater play to the positive role of social security in building a new network of rural social relations. For rural areas, the government should increase support for social security, find a proper balance in terms of economic and public resources. For the central and western regions, it should also strengthen the supervision of rural support funds, improve the utilization rate of funds, and ensure that rural social security policies are implemented. For the eastern region, it should increase transfer payments to the central and western regions to make up for the financial gap in the field of social security in regions. In addition, the eastern regions should summarize effective measures and advanced experiences, and share them with the central and western regions.

Our study fills an important gap in the literature by investigating the impacts of social insurance on social interactions. However, due to the limitations of data, there are still many aspects which could be future research directions. Firstly, this study takes the data of Chinese General Social Survey in 2013 as samples. If we get further years’ data, we may be able to draw richer conclusions and policy recommendations. Secondly, social insurance can affect social interactions via various mechanism. If we can obtain corresponding data, we can test it and provide empirical evidence. Thirdly, there are lots of factors which will exert impact on social interactions, we may get more robust results if our models can control these variables.

## Data Availability Statement

Publicly available datasets were analyzed in this study. This data can be found here: https://chfs.swufe.edu.cn.

## Author Contributions

MZ, LY, and JJ conceived and designed the outline. MZ, ZK, HT, and GL performed the study. All authors contributed to the article and approved the submitted version.

## Conflict of Interest

The authors declare that the research was conducted in the absence of any commercial or financial relationships that could be construed as a potential conflict of interest.

## Publisher’s Note

All claims expressed in this article are solely those of the authors and do not necessarily represent those of their affiliated organizations, or those of the publisher, the editors and the reviewers. Any product that may be evaluated in this article, or claim that may be made by its manufacturer, is not guaranteed or endorsed by the publisher.
